# Novel Analytical Methods in Food Analysis

**DOI:** 10.3390/foods11101512

**Published:** 2022-05-23

**Authors:** Philippe Delahaut, Riccardo Marega

**Affiliations:** Analytical Laboratory, CER Groupe, Rue du Point du Jour 8, 6900 Marloie, Belgium; r.marega@cergroupe.be

Food analysis is a discipline with a huge impact on both economical and medical aspects of modern societies, meaning that it is at the cornerstone between industrial, medical, and regulatory needs.

The development of analytical methods in food matrices has always been difficult due to the large variety of their physicochemical properties (e.g., physical state, lipid content, pH, among others), which can change analyte structure and extraction efficiencies (e.g., Mallard reactions) due to different processing throughout preparation and distribution (e.g., fermentation, heating, mechanical stress).

On the one hand, such complexity can be tackled by a combination of sample preparation protocols and use of analytical instrumentation that is typically available in specialized laboratories (in terms of personnel and equipment, e.g., mass spectrometry analysis of food allergens). On the other hand, there is a great demand for the “decentralization” of analytical food methods by means of protocol simplification and on-site analysis (e.g., portable immunoassays). Furthermore, the simultaneous detection of multiple analytes at the same time (multiplexing) is an ongoing trend in the development of methods and instruments that increase throughput while lowering costs and operator intervention.

Integration of biological reagents (antibodies, aptamers), materials (nanoparticles, nanotubes), technologies (microspotting, microfluidics), and physical principles (spectroscopy and spectrometry) is today consolidating at both the academic and industrial levels, aiming at the exploration and control of the vast chemical space intersecting with food analysis.

Marchand et al. propose a strategy combining non-targeted and targeted lipidomics MS-based approaches to identify disrupted patterns in serum lipidome upon growth promoter treatment in pigs [1]. Evaluating the relative contributions of the platforms involved, the study aims at investigating the potential of innovative analytical approaches to highlight potential chemical food safety threats. The strategy enabled highlighting specific lipid profile patterns involving various lipid classes, mainly in relation to cholesterol esters, sphingomyelins, lactosylceramide, phosphatidylcholines, and triglycerides. Thanks to the combination of non-targeted and targeted MS approaches, various compartments of the pig serum lipidome could be explored, including commonly characterised lipids (by Lipidyzer™ platform kits), triglyceride isomers (by triglyceride platform methods) and unique lipid features (by non-targeted LC-HRMS). Thanks to their respective characteristics, the complementarity of the three tools could be demonstrated for public health purposes, with enhanced coverage, level of characterization, and applicability.

Pietschmann et al. discuss how the misuse of antibiotics as well as incorrect dosage or insufficient time for detoxification can result in the presence of pharmacologically active molecules in fresh milk [2]. Hence, in many countries, commercially available milk has to be tested with immunological, chromatographic, or microbiological analytical methods to avoid consumption of antibiotic residues. They thus report on a novel, sensitive and portable assay setup for the detection and quantification of penicillin and kanamycin in whole fat milk (WFM) based on competitive magnetic immunodetection (cMID). Their results demonstrate the suitability of cMID-based competition assay for reliable and easy on-site testing of milk.

Damiani et al. report on the origin discrimination of Argentinian honeys as a case study to compare the capabilities of three spectroscopic techniques as fast screening platforms for honey authentication purposes [3]. Each sample was fingerprinted by FT-MIR, NIR and FT-Raman spectroscopy. The results obtained in their work suggests the major potential of FT-MIR for fingerprinting-based honey authentication and demonstrate that accuracy levels that may be commercially useful can be reached.

Kuragano et al. developed a microliter-scale high-throughput screening (MSHTS) system for Aβ42 aggregation inhibitors using quantum-dot nanoprobes [4]. This study aimed at elucidating whether the MSHTS system could be applied to the evaluation of processed foods. Therefore, they examined Aβ42 aggregation inhibitory activity of salad dressings, including soy sauces. They demonstrated that non-heat-treated raw soy sauce exhibited higher Aβ42 aggregation inhibitory activity than heat-treated soy sauce, and concluded that MSHTS system can be applied to processed foods.

Schelm et al. report on the development of methods for detecting possible adulterations on truffles [5]. A real-time PCR (polymerase chain reaction) assay allowing the detection and quantitation of Asian black truffles in Tuber melanosporum up to 0.5% was developed. In addition, a capillary gel electrophoresis assay was designed, which allows for the identification and quantitation of different species. The methods can be used to ensure the integrity of truffle products.

Jafari et al. discuss in their review the increasing demand for portable and handheld devices to provide rapid, efficient, and on-site screening of food contaminants [6]. Recent technological advancements in the field include smartphone-based, microfluidic chip-based, and paper-based devices integrated with electrochemical and optical biosensing platforms. Furthermore, the potential application of portable mass spectrometers in food testing might bring (in the future) the confirmatory analysis from the laboratory to the field. To this end, the analytical performance of these devices and the extent they match the World Health Organization benchmark for diagnostic tests (i.e., the Affordable, Sensitive, Specific, User-friendly, Rapid and Robust, Equipment-free, and Deliverable to end-users (ASSURED) criteria) was evaluated critically. A five-star scoring system was used to assess their potential to be implemented as food safety testing systems. The main findings highlight the need for concentrated efforts towards combining the best features of different technologies, to bridge technological gaps and meet commercialization requirements.

Bergwerff et al. discuss in their review how food microbiology is deluged by a vastly growing plethora of analytical methods [7]. The context is that the highest risk of food contamination comes through the animal and human fecal route, with a majority of foodborne infections originating from sources in mass and domestic kitchens at the end of the food-chain. Whatever the scientific and technological excellence and incentives, the decision-maker determines this implementation after weighing mainly costs and business risks.

Gavage et al. report on the recent accessibility and technological improvements of high resolution mass spectrometry (HRMS) for the analysis of different classes of contaminants and residues [8]. This kind of instrument is often considered as a research tool, but the wide range of potential contaminants and residues that must be monitored, is increasing. Their review aims, through a series of relevant selected studies and developed methods dedicated to the different classes of contaminants and residues, to demonstrate that HRMS can reach detection levels in compliance with current legislation and is a versatile and appropriate tool for routine testing.

Tsagkaris et al. critically review the available screening methods for pesticide residues based on optical detection during the period 2016–2020 [9]. Optical biosensors are commonly miniaturized analytical platforms introducing the point-of-care (POC) era in the field. Various optical detection principles have been utilized, namely colorimetry, fluorescence (FL), surface plasmon resonance (SPR), and surface enhanced Raman spectroscopy (SERS). Overall, despite being in an early stage facing several challenges (i.e., long sample preparation protocols or interphone variation results), such POC diagnostics pave a new road into the food safety field in which analysis costs will be reduced and a more intensive testing will be achieved.

Walpurgis et al. propose a narrative review with an overall aim of indicating the current state of knowledge and the relevance concerning food and supplement contamination and/or adulteration with doping agents and the respective implications for sportspeople drug testing [10]. The identification of a doping agent (or its metabolite) in sports drug testing samples constitutes a violation of the anti-doping rules defined by the World Anti-Doping Agency. Reasons for such adverse analytical findings (AAFs) include the intentional misuse of performance-enhancing/banned drugs. While the sensitivity of assays employed to test pharmaceuticals for impurities is in accordance with good manufacturing practice guidelines allowing to exclude any physiological effects, minute trace amounts of contaminating compounds can still result in positive doping tests. In addition, food was found to be a potential source of unintentional doping, the most prominent example being meat tainted with the anabolic agent clenbuterol.

The research manuscripts and reviews reported in this special issue are thus representative examples of the complexity, variety and demands of the food analysis domain. Indeed, spectrometry-based methods (mass, vibrational) remain the reference ones for laboratory-scale analytical demands, while immunoassays are still the most common base for portable assay development for on-site applications. While laboratory scale methods are gaining sensibility and operability thanks to hardware and software optimizations (data-treatment and analysis), portable ones are exploring the use of more alternative signal transducers (e.g., nanoparticles) and on the integration of the analytical result with the data intrinsically originating from hand-held devices (automated time, localization and cloud based analytical data transmission). At last, it has to be noted how methods that were initially designed to reply a specific analytical request are then used to provide answers to fields that are different from the original ones. In this respect, the examples reported herein concerning how to check the lipidome profile in order to detect β-agonist use in animals and on how to relate dietary consumption with athlete’s scores after anti-doping test are explanatory of the current trend of scope broadening and wide applicability of some methods. Taken together, these manuscripts highlight how the creativity of the authors in particular, and of the scientific community in general, is yielding novel analytical methods and responses to longstanding problems in food analysis.

## Data Availability

Not applicable.

## References

[1] Marchand J., Guitton Y., Martineau E., Royer A.-L., Balgoma D., Le Bizec B., Giraudeau P., Dervilly G. (2021). Extending the Lipidome Coverage by Combining Different Mass Spectrometric Platforms: An Innovative Strategy to Answer Chemical Food Safety Issues. Foods.

[2] Pietschmann J., Dittmann D., Spiegel H., Krause H.-J., Schröper F. (2020). A Novel Method for Antibiotic Detection in Milk Based on Competitive Magnetic Immunodetection. Foods.

[3] Damiani T., Alonso-Salces R.M., Aubone I., Baeten V., Arnould Q., Dall’Asta C., Fuselli S.R., Pierna J.A.F. (2020). Vibrational Spectroscopy Coupled to a Multivariate Analysis Tiered Approach for Argentinean Honey Provenance Confirmation. Foods.

[4] Kuragano M., Yoshinari W., Lin X., Shimamori K., Uwai K., Tokuraku K. (2020). Evaluation of Amyloid Β42 Aggregation Inhibitory Activity of Commercial Dressings by A Microliter-Scale High-Throughput Screening System Using Quantum-Dot Nanoprobes. Foods.

[5] Schelm S., Siemt M., Pfeiffer J., Lang C., Tichy H.-V., Fischer M., Kuragano M., Yoshinari W., Lin X., Shimamori K. (2020). Food Authentication: Identification and Quantitation of Different Tuber Species via Capillary Gel Electrophoresis and Real-Time PCR. Foods.

[6] Jafari S., Guercetti J., Geballa-Koukoula A., Tsagkaris A.S., Nelis J.L.D., Marco M.-P., Salvador J.-P., Gerssen A., Hajslova J., Elliott C. (2021). ASSURED Point-of-Need Food Safety Screening: A Critical Assessment of Portable Food Analyzers. Foods.

[7] Bergwerff A.A., Debast S.B. (2021). Modernization of Control of Pathogenic Micro-Organisms in the Food-Chain Requires a Durable Role for Immunoaffinity-Based Detection Methodology—A Review. Foods.

[8] Gavage M., Delahaut P., Gillard N. (2021). Suitability of High-Resolution Mass Spectrometry for Routine Analysis of Small Molecules in Food, Feed and Water for Safety and Authenticity Purposes: A Review. Food.

[9] Tsagkaris A.S., Pulkrabova J., Hajslova J. (2021). Optical Screening Methods for Pesticide Residue Detection in Food Matrices: Advances and Emerging Analytical Trends. Foods.

[10] Walpurgis K., Thomas A., Geyer H., Mareck U., Thevis M. (2020). Dietary Supplement and Food Contaminations and Their Implications for Doping Controls. Foods.

